# Medical journal requirements for clinical trial data sharing: Ripe for improvement

**DOI:** 10.1371/journal.pmed.1003844

**Published:** 2021-10-25

**Authors:** Florian Naudet, Maximilian Siebert, Claude Pellen, Jeanne Gaba, Cathrine Axfors, Ioana Cristea, Valentin Danchev, Ulrich Mansmann, Christian Ohmann, Joshua D. Wallach, David Moher, John P. A. Ioannidis

**Affiliations:** 1 Univ Rennes, CHU Rennes, Inserm, CIC 1414 [(Centre d’Investigation Clinique de Rennes)], Rennes, France; 2 Meta-Research Innovation Center at Stanford (METRICS), Stanford University, California, United States of America; 3 Department for Women’s and Children’s Health, Uppsala University, Uppsala, Sweden; 4 Department of Brain and Behavioral Sciences, University of Pavia, Pavia, Italy; 5 Stanford Prevention Research Center, Stanford University School of Medicine, Stanford, California, United States of America; 6 Ludwig-Maximilians University Munich, Institute for Medical Information Processing, Biometry, and Epidemiology, München, Germany; 7 Ludwig-Maximilians University Munich, OSCLMU—Open Science Center LMU, München, Germany; 8 European Clinical Research Infrastructure Network (ECRIN), Düsseldorf, Germany; 9 Department of Environmental Health Sciences, Yale School of Public Health, New Haven, Connecticut, United States of America; 10 Center for Journalology, Clinical Epidemiology Program, Ottawa Hospital Research Institute, Ottawa, Canada; 11 School of Epidemiology and Public Health, University of Ottawa, Ottawa, Canada; 12 Departments of Medicine, of Epidemiology and Population Health, of Biomedical Data Science, and of Statistics, Stanford University, California, United States of America

## Abstract

Florian Naudet and co-authors discuss strengthening requirements for sharing clinical trial data.

**Summary points**

- Efficient sharing and reuse of data from clinical trials are critical in advancing medical knowledge and developing improved treatments.
- We believe that the International Committee of Medical Journal Editors (ICMJE) clinical trial data sharing policy is currently inadequate.
- Although data sharing plans help increase transparency, they do not ensure that data are shared, and they are often inadequately implemented.
- We believe that the ICMJE should adapt a stronger policy on data sharing that is enforced rigorously in all ICMJE members and affiliated journals.
- The policy should include a strong evaluation component to ensure that all clinical trial data are shared, their value maximized, and data producers incentivized.

In some science, technology, engineering, and mathematics (STEM) fields, data sharing is the norm (e.g., physics or space science). However, this is currently not the case in biomedicine, except for certain exceptions in areas such as genomics. For therapeutic research, data sharing is expected to maximize the value of research for clinical practice by means of greater transparency and opportunities for external researchers to reanalyze, synthesize, replicate, and build upon previous evidence. Examples include reanalyses, secondary analyses, individual patient data (IPD) meta-analyses, and methodological evaluations. Maximizing the efficient use of clinical research data is important in the development of new therapeutic options, including treatments for the Coronavirus Disease 2019 (COVID-19).

However, despite the prominent role of clinical trial data in evidence-based medicine, data sharing in clinical trials was almost nonexistent even years after the United States National Institutes of Health (NIH) recognized its value in their 2003 Data Sharing Policy [1]. In the past 10 years, several initiatives leading to research infrastructures (repositories) have been launched to promote data sharing, including data repositories such as the Clinical Study Data Request [CSDR] and the Yale University Open Data Access [YODA]. Overall, there has been a lack of effective policies to ensure that study data are maximally available and reusable: Certain journals require data sharing, but their guidelines have been inconsistent and unclear [2]. A minority of publishers or journals, such as PLOS and the *British Medical Journal* (*BMJ*), have stronger data sharing requirements [3].

Several influential groups have developed data sharing guidance and policies. In 2016, the International Committee of Medical Journal Editors (ICMJE, which currently has 14 journal and organizational members) published a proposal [4] stating that there is an ethical obligation toward trial participants who have volunteered and put themselves at risk to help generate information about the safety and efficacy of interventions, to responsibly share clinical trial data. The ICMJE suggested that deidentified IPD should be made publicly available no later than 6 months after publication of the main trial results. However, this proposal triggered debate, and many investigators have expressed skepticism toward this proposal [5]. The concerns raised include the feasibility of the proposed requirements for data sharing, the resources needed, the real or perceived risks for trial participants, and the need to protect the interests of patients and researchers. Some of those concerns were found to be unwarranted. For example, survey evidence suggests that patients are willing to have their data shared in a responsible and secure manner [6]. Nevertheless, the ICMJE moderated their initial proposal. Their final requirements did not make data sharing mandatory, but required a data sharing plan to be included in each paper from July 1, 2018 and prespecified in study registration for clinical trials beginning enrollment of participants after January 1, 2019, as a condition for publication [7]. However, outputs of the Reproducibility in Therapeutic Research program suggest that this policy is unlikely to be met with current practices [8–10]. Although a data sharing plan is not enough to ensure that data are shared [3], even this basic, first step is insufficiently implemented.

## Performance of the ICMJE data sharing policy

Journals considered by the ICMJE as affiliated journals (i.e., journals stating that they follow the ICMJE recommendations) are not mandated by the ICMJE to respect the ICMJE policy, and there is no ICMJE data sharing dashboard to monitor data sharing activities. The ICMJE clearly states that ICMJE “cannot verify the completeness or accuracy” of the list of affiliated journals (see the ICMJE website: http://www.icmje.org/journals-following-the-icmje-recommendations). Indeed, there is suboptimal implementation of ICMJE data sharing requirements among the ICMJE member journals themselves. For instance, in 2019 (at this time there were 14 member journals), 8/14 had an explicit data sharing policy (including 3 more stringent and 1 less stringent than the ICMJE requirements); 5/14 had a statement that they followed ICMJE requirements without further details; and 1/14 had no policy on their website [8]. Additionally, the ICMJE website outlines that many affiliated journals may not follow their recommendations. In addition, despite some uncertainty regarding the definition of predatory journals, evidence suggests that around 30% of affiliated journals are potentially predatory journals [11], with editorial practices that “deviate from best editorial and publication practices” [12]. It is unlikely that such journals have any effective data sharing policy in place.

In an analysis of 489 randomly selected ICMJE-affiliated journals that published a randomized controlled trial (RCT) in 2018, with an accessible online website and not considered as potentially predatory journals, 30% (95% confidence interval [CI]: 26% to 34%) had an explicit data sharing policy on their website [8]. Of these, 7% were more stringent, 59% were less demanding, and 34% were compliant with the ICMJE policy. Furthermore, 56% of the sample (95% CI: 52% to 61%) only referred to ICMJE requirements, and 14% (95% CI: 11% to 17%) had no data sharing policy, and their instructions did not allude to the ICMJE recommendations.

In a random sample of 100 articles on RCTs published in 2019 in the 14 ICMJE member journals, there were data sharing statements in 98% (95% CI: 92% to 99%). However, data sharing statements can simply state that “no data are available” [8]. An intention to share IPD was expressed in 77% (95% CI: 67% to 85%) of articles. However, in a random sample of 100 articles published in 2019 on RCTs published in ICMJE-affiliated journals with a data sharing policy, there were data sharing statements in only 25% (95% CI: 17% to 35%), and intention to share IPD was expressed in 22% (95% CI: 15% to 32%).

According to an evaluation of 487 RCTs with a data sharing statement published in *JAMA*, *The Lancet*, and *NEJM* between July 1, 2018 and April 4, 2020, only 2 (0.5%) of the 334 RCTs declaring they shared data had deidentified IPD available on the journal website that could be downloaded and reused. Another 89 (27%) articles proposed to store data in repositories, but data were stored for only 17 studies, mostly because of restrictions due to embargo periods pending product approval. The remainder were described as being accessible via request to a committee, authors, or company and unspecified for 15 (5%) [13].

While data sharing statements are an important first step, a promise to share data does not guarantee that data will be made available when requested. A previous analysis of RCT articles published in *BMJ* and *PLOS Medicine*, 2 prominent medical journals with a policy requiring RCT data sharing (since January 2013 for RCTs on drugs and devices and July 2015 for all therapeutics for *BMJ*, and after March 2014 for all types of interventions for *PLOS Medicine*) found that for only 46% (95% CI: 30% to 62%) of the eligible studies had the original investigators shared their data with sufficient information to enable reanalysis [3]. For trials submitted and published subsequent to the adoption of data sharing policies by these journals, 24% of deidentified IPD were retrievable for downloading and use, 65% were declared available upon request, 3% were embargoed, and 8% were declared not available.

The ICMJE also requires data sharing plans for registered trials: “clinical trials that begin enrolling participants on or after 1 January 2019 must include a data-sharing plan in the trial registration” [7]. Indeed, there are certain important issues (such as those related to informed consent) that should ideally be prespecified and fixed before the trial is started to make data sharing possible in practice. However, here, the implementation of the policy is worse, even for trials supported by funders with data sharing policies promoting IPD sharing. A 2019 survey of RCTs registered on ClinicalTrials.gov by funders with a data sharing policy found that data sharing plans were present for 77% (95% CI: 67% to 84%) and 81% (95% CI: 72% to 88%) of RCTs funded by noncommercial and commercial funders, respectively [9]. An expressed intention to share data was found in 12% (95% CI: 7% to 20%) and 59% (95% CI: 49% to 69%) of RCTs funded by noncommercial and commercial funders, respectively.

## Beyond data sharing to data reuse

Although data availability is a prerequisite for effective reuse of the data, it is actual reuse of data that leads to the expected benefits of clinical trial data sharing. However, actual reuse of data is difficult to gauge. The *Annals of Internal Medicine* has encouraged, although not required, data sharing since 2007, but over the subsequent decade, articles expressing an intention to share data were not associated with published reuse of data (i.e., reanalysis, secondary analysis, or IPD meta-analysis) [10]. Arguably, authors who commit to data sharing may in fact be reluctant to share their data. They may be insufficiently incentivized for data sharing, as the current evaluation of scientists relies on traditional criteria (e.g., productivity in terms of authored publications) as opposed to progressive criteria (e.g., data sharing) [14]. Furthermore, authors are often difficult to contact and may lack the time, knowledge, technical infrastructure, or financial resources to prepare and share the data sets [3]. In addition, authors may not be aware of the resources available to facilitate data sharing and may face legal difficulties in sharing their data, as the definition of “anonymization” is not universal (e.g., in the European context), can be ambiguous, and carries a risk of loss of information [15].

It is also possible that data are not requested; indeed, usage metrics from repositories such as YODA and CSDR suggest that a large majority of data is not requested or that reuses may remain unpublished. Practical issues could contribute to this problem, as the process of data sharing on these platforms is still cumbersome, and data shared without codes or proper dictionaries may be impossible to reanalyze and reuse. An alternative possibility is that many trials where data become available are small or poorly designed studies presenting little interest for reuse of their data, while large, pivotal trials often fail to offer data sharing. For instance, the Data Ark initiative was unable to retrieve data from the majority of the most widely cited studies published between 2006 and 2016 in the fields of psychology and psychiatry [16]. Furthermore, since various data sharing platforms are siloed, reuse is currently difficult to measure systematically, preventing the evaluation of its benefits.

## A call for action: Toward policies and practices that maximize clinical trial value

These discouraging findings call for action in order to ensure that the ICMJE data sharing policy will draw nearer to its goal of “creating an environment in which the sharing of de-identified individual participant data becomes the norm” [7]. Realistically, we recognize that the ICMJE cannot achieve this change alone without support and endorsement by other stakeholders. We call for the ICMJE to take the lead in charting a path toward policies and practices that maximize clinical trial value. This path could then lead to a cultural shift toward enhanced data sharing, perhaps supported by institutions, funders, and others. While journals are the custodians of research articles, there appears to be little attention to the expressed desires of patients, surely equally important players in knowledge dissemination. We see an urgent need for data sharing policies to be strengthened, adequately implemented, and monitored. Secondly, easy-to-use technical infrastructures, administrative processes, and practice guidelines are needed for successful implementation of the different policies. Lastly, clinical trial data sharing should be adequately identified, recognized as a behavior that increases research integrity, and therefore incentivized in line with the Hong Kong Principles for assessing researchers [17]. There is a need to reach a consensus on the best practices for data generators, curators, and reusers in data sharing. Rewarding and recognizing adequately these best practices are expected to be paramount in increasing data sharing value. In addition, any incentives must preemptively consider how to diminish, rather than exacerbate, the inequities between developed and developing countries in generation and use of data [18].

A centralized approach toward monitoring of data sharing is needed, where data sharing dashboards would collect these data sharing metrics (e.g., intention to share, data sharing, and data reuse) from journals, funders, and repositories and then present it to the research community. Currently, this information is siloed (e.g., each data sharing platform presents this information separately) and is not presented broken down by journal and by specific journal articles. Recent efforts have been made to monitor data sharing, and other open science practices, in journal articles [19], and these tools (https://github.com/quest-bih/oddpub) are already used at the QUEST center for transforming biomedical research in Berlin, Germany. These initiatives need to be generalized and be included in ICMJE policy. The challenges identified, together with suggested changes to ICMJE policy and details of the necessary evaluations, are listed in **Table 1**.

**Table 1:** Some identified challenges, suggestions, and evaluation components for the ICMJE data sharing policy.

Identified challenge	Suggested change to the ICMJE policy	Evaluation component
Poor implementation of the policy by ICMJE-affiliated journals.	To certify ICMJE-affiliated journals based on their implementation of the policy. This could be facilitated if journals have a reproducibility research editor.	Developing software to monitor journals’ implementation of ICMJE policy, e.g., in line with the TOP factor developed by the Center for Open Science.
Suboptimal intention to share data by RCTs published in ICMJE journals with a data sharing policy for RCTs.	Policies should require data sharing unless major obstacles exist.	Monitoring ICMJE-affiliated journals’ enforcement of the policy by implementing software to check whether papers offer data sharing, similar to that proposed by the Berlin QUEST center.
Suboptimal intention to share data by RCTs in clinical trial registration on databases such as ClinicalTrials.gov for funders with a data sharing policy for RCTs.	Policies should require the use of registries making intention to share data mandatory.	Monitoring compliance with funders/sponsors’ policies by implementing software to check whether data sharing plans offer data sharing and reporting of this information by funders/sponsors, e.g., Trial Tracker for clinical trial results and the Good Pharma Scorecard (https://bioethicsinternational.org/good-pharma-scorecard/) for pharmaceutical firms.
“Data sharing upon request” is not sufficient to ensure that data are shared.	Policies should favor data deposition when it is ethically possible. Policies should also outline more clearly the procedures that data requesters should follow and how journals can reinforce data sharing in case of noncompliance with promises.	Monitoring data availability by implementing practical tests of the policy. Performing interventional studies to evaluate mechanisms of sanction and incentives.
Impact of clinical trial data sharing is still insufficiently documented.	State explicitly that policy aiming to reform medical science needs to be evidence based. Policy should be continuously informed and revised via a strong evaluation component.	Defining and testing best practices in clinical trial data sharing to maximize clinical trial value. Prospectively monitoring the impact of data sharing policies on the progress of medical research, using observational and interventional designs. This implies developing a tool to identify clinical trial data reuse and then to track the impact of reuses. Portals are needed that collect this type of data from a wide range of sources (journals, funders, repositories …) since currently, all this information is siloed.

ICMJE, International Committee of Medical Journal Editors; RCT, randomized controlled trial.

We argue that the current practice of journals listed as ICMJE affiliated without committing to its policies undermines the value of the affiliation. A basic certification reflecting policy implementation could increase commitment. For example, the “TOP factor,” an indicator dedicated to assessing transparency, openness, and reproducibility (https://www.topfactor.org/), could provide a certification of this nature. Specifically, a TOP factor of 3 indicates that the policy includes both a requirement and a verification process for the correspondence of data with the findings reported in the paper. For this purpose, each journal could have a Reproducible Research Editor, like *the Biometrical Journal*, for instance, with specific infrastructures to submit data and analyze manuscripts provided on the journal’s web-based submission platform. Beyond journal metrics, article-based indicators can also be used to explore enforcement of the policy. Funder-based metrics like the “Good Pharma Scorecard” [20] could also help improve research transparency. Together, such initiatives can be seen as primary steps in incentivizing data sharing behaviors.

Currently, the ICMJE policy only encourages data sharing but does not guarantee it. A more binding policy to favor data deposition whenever ethically possible is needed. We believe that the policy should include adequate incentives for data sharing, as part of hiring, promotion, and tenure of researchers, together with reinforcement measures that journals can adopt in case of noncompliance with data sharing requirements. Incentives and sanctions should be implemented and evaluated to see if intentions are achieved. It would be unrealistic to expect all journals to endorse the same incentives and the same sanctions. Moreover, it should not be taken for granted that any incentive and any sanction would work. Specific interventions can be piloted at the level of single journals or groups of journals that feel comfortable with implementing and evaluating these policies. Interventions need to be evidence based. For instance, it has been suggested that awarding badges for data sharing could be an efficient incentive for data availability. Still, randomized evidence suggested that these tools may be ineffective in the area of biomedical science [21]. Even more challenging, the evidence gathered to inform the policy should not be limited to surrogate indicators such as data availability, but should also assess whether the data are really used and whether these reuses have an impact in moving medical research forward faster, by exploring data sharing benefits and its possible limitations, information that is currently lacking. To this end, tracking reuses and the impact of reuses could be a good starting point.

This agenda requires changes to the ICMJE policy itself and also coordinated efforts by various stakeholders such as researchers, journals, funders, and institutions, as illustrated in **Table 2**. It implies joining forces in an observatory of clinical data sharing practices with continuous monitoring of journal outputs and empirical evaluations to measure the value of the ICMJE data sharing requirements. Greater consideration and rewarding of best practices in data sharing can help incentivize data generators, particularly those who work in low-income countries [18]. At a more global level, it will provide necessary feedback on the ICMJE data sharing policy and could indicate any action that might be needed to increase the value of clinical trial data sharing. Ultimately, our ethical obligation to clinical trial participants is to optimally use the data gathered to achieve improved clinical outcomes and thereby benefit human health.

**Table 2:** Proposed actions for various stakeholders to ensure that the ICMJE policy meets the mark.

Stakeholders	Proposed action
**ICMJE**	Should certify compliance, adopt more binding policies, and clarify when clinical trial data sharing is required and ethically possible.
**Journals**	Should provide oversight with editorial screening (e.g., by a reproducible research editor) and software screening (e.g., by implementing an IT infrastructure to verify data sharing processes described in submitted data sharing plans). Should embargo future publications from authors if they have not shared their data from previous manuscripts in their journal despite a promise to do so.
**Funders/institutions**	Should monitor and reward data sharing. Should provide technical/regulatory guidance for clinical trial data sharing. Should implement DUACs. Should withhold support from investigators not sharing data. Should support meta-research efforts that evaluate the impact of clinical trial data sharing.
**Researchers**	Should commit to sharing data. Should engage in evaluating the impact of clinical trial data sharing and provide the necessary feedback to improve the policy.

DUAC, Data Use and Access Committee; ICMJE, International Committee of Medical Journal Editors.

## Abbreviations

*BMJ*: *British Medical Journal*
CI: confidence interval
COVID-19: Coronavirus Disease 2019
CSDR: Clinical Study Data Request
ICMJE: International Committee of Medical Journal Editors
IPD: individual patient data
NIH: National Institutes of Health
RCT: randomized controlled trial
STEM: science, technology, engineering, and mathematics
YODA: Yale University Open Data Access
